# Reversed Three-Dimensional Visible Light Indoor Positioning Utilizing Annular Receivers with Multi-Photodiodes

**DOI:** 10.3390/s16081254

**Published:** 2016-08-08

**Authors:** Yinfan Xu, Jiaqi Zhao, Jianyang Shi, Nan Chi

**Affiliations:** Key Laboratory for Information Science of Electromagnetic Waves (MoE), Department of Communication Science and Engineering, Fudan University, No. 220, Handan Road, Shanghai 200433, China; xuyinfan_tx@126.com (Y.X.); 15210720132@fudan.edu.cn (J.Z.); 14110720025@fudan.edu.cn (J.S.)

**Keywords:** reversed positioning, VLC, multi-photodiodes, angle, RSS

## Abstract

Exploiting the increasingly wide use of light emitting diodes (LEDs) lighting, in this paper we propose a reversed indoor positioning system (IPS) based on LED visible light communication (VLC) in order to improve indoor positioning accuracy. Unlike other VLC positioning systems, we employ two annular receivers with multi-photodiodes installed on the ceiling to locate the persons who carry LEDs. The basic idea is using multi-photodiodes to calculate the angle while using the received signal strength (RSS) method to calculate the distance. The experiment results show that the effective positioning range of the proposed system is 1.8 m when the distance between two receivers is 1.2 m. Moreover, a positioning error less than 0.2 m can be achieved under the condition that the radius of the PIN circle is between 0.16 m and 0.2 m, and the distance of the transmitter-receiver plane is less than 1.8 m, which will be effective in practice.

## 1. Introduction

Recently, visible light communication has been widely researched and it attracts tremendous attention due to the growing global market of LEDs. Widespread used, cost effectiveness, high brightness, and larger bandwidth make it the most promising candidate for communications, especially in some specific areas such as hospitals, aircraft, underwater and high-security-requirement environments [1]. In addition, this green, energy-saving communications technology has lately been developed in the application of IPS. GPS has largely solved the problem for outdoor scenarios [2]. However, because of strong attenuation and shadowing effects, GPS does not work in indoor environments [3]. Research into IPS has been performed using radio frequency, infrared, ultrasound, and wireless local area networks (WLAN), though these systems are also limited due to factors such as the presence of additional infrastructure, large measurement error, narrow tracking range, electromagnetic interference, low security, long response time, and low scalability [4,5]. In addition, these systems have low scalability because signal interference can occur when the number of users increases and because they require investment in infrastructure [6]. Compared with the previous positioning techniques, indoor positioning technology based on the VLC system has a broad development prospect due to its high accuracy, immunity to electromagnetic interference and low power consumption. As a result, the visible light–based positioning system is receiving attention because the system is more accurate and has well-equipped infrastructure [7].

A number of papers have described the use of LEDs for localization [8]. VLC-based IPS can be generalized into two formats: photodiode-based (PD-based) positioning and image sensor–based positioning [9]. In PD-based systems, the distance of a receiver is estimated based on a measurement in the various forms of received signal strength (RSS) information, angle of arrival (AOA), time of arrival (TOA), time difference of arrival (TDOA), and so on [10]. According to Reference [11], the RSS positioning algorithm can achieve an accuracy of 7 cm. The TDOA system has the maximum location error of 4.5 mm with an average location error of 1.8 mm. The RSS method is less immune to noise and less accurate as compared to the TDOA and TOA methods. However, the TOA and TDOA methods require synchronization, which will increase their complexity and high performance. The image positioning system consists of a LED array and an imaging receiver with multiple detectors. Each LED in a lighting fixture sends a differential three-dimensional space coordinate, and an image sensor receives the signals. The scheme uses a collinearity condition to relate the three-dimensional space coordinate data of the LEDs to the two-dimensional coordinate data of the image sensor [12]. However, the image processing is so complicated that it requires additional image processing techniques and limits the data rate.

In prior works, the height of the receiver is known so that the coordinates on the horizontal plane can be calculated [13]. In this paper, assuming the height is unknown, we propose a reversed 3D VLC IPS using a single transmitter and two annular receivers with four PINs respectively. In addition, a novel hybrid positioning technology is proposed, which uses multi-photodiodes to calculate the angle and uses the RSS method to calculate the distance. Compared with traditional VLC positioning systems, the function and application scenario of the proposed system are different. The main function of prior VLC positioning systems is providing users with their own precise location. This kind of system is suitable for navigation in shopping malls, airports and other large buildings. In addition, the ultimate goal of the proposed system is to realize personnel monitoring of some large electromagnetic-sensitive sites, such as soldiers in aircraft carriers and workers in nuclear power stations. The control centers can ensure the safety of the personnel by constantly monitor the staff's exact locations. Furthermore, instead of updating the LEDs by changing the structure, the proposed reversed positioning system only needs a few receivers, which is more convenient and cost-efficient. Besides, LEDs can be embedded into the helmets worn by workers and shine upward. So it will avoid an uncomfortable visual experience for the users. By adopting discontinuous transmitting technology, the battery life can exceed 20 h in practical applications. The above technical advantages make the proposed reversed VLC-based IPS become a relatively suitable and effective solution in the case of nuclear power stations and aircraft carriers.

## 2. Materials and Methods

**Figure 1:**
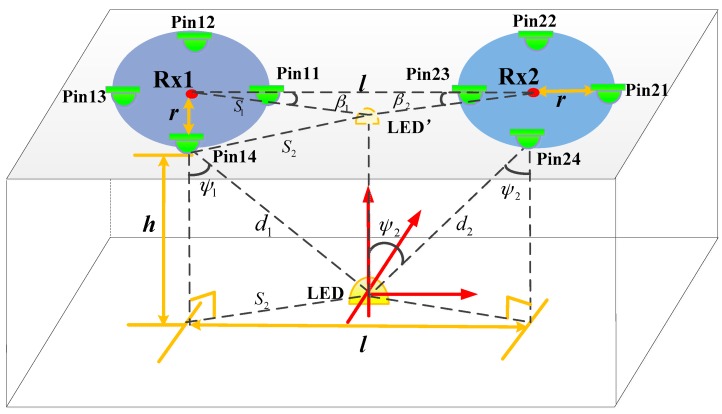
Schematic of VLC indoor positioning system.

As shown in Figure 1, in the proposed VLC positioning system, RX1 and RX2 are two annular receivers with four evenly distributed PINs, which are installed on the ceiling. People on the ground carry LEDs on their heads to send positioning signal as transmitters. In order to get the 3D location information of people, we adopt the hybrid positioning algorithm by combining the distance with angles. Since the distances and angles are related to the receiving light intensity, we can calculate the distances on the basis of intensity and obtain the angles according to the intensity difference between different PINs.

The luminous intensity of LEDs agrees with Lambert's cosine law, and it is assumed that only one Lambert luminous mode exists in an LED chip. Regardless of the air and device refraction, we consider that radiation angle φ equals the incidence angle ψ. The received optical power $P_{r}$ at a receiver in a direct link can be represented in Equation (1) [6,7,8,9]:

(1) $$P_{r}=\frac{(m+1)A}{2\pi d^{2}}\cdot \frac{n^{2}}{\sin^{2}\psi_{c}}\cos^{m+1}(\psi )Ts(\psi )P_{t}$$

where m represents the Lambertian order; d is the distance between the LED and the receiver; A is the physical area of a photodiode; n is the refractive index of the optical concentrator; $\psi_{c}$ is the incidence angle of the light with respect to the normal vector of a photodiode; Ts(ψ) is the gain of the optical filter; and $P_{t}$ is the average transmitted optical power [10,11]. All the constants are expressed by formula C as follows:

(2) $$C=\frac{(m+1)n^{2}A}{2\pi \sin^{2}\psi_{c}}\cdot Ts(\psi )$$

Thus, the received optical power can be described as Equation (3):

(3) $$P_{r}=\frac{C}{d^{2}}\cdot \cos^{m+1}(\psi )P_{t}$$

For typical LED bulbs with limited illumination ranges such as ±60°, we have *m* = 1 [12]. In Figure 1, the radius of the annular receiver’s circle is r; the distance between two receivers is l; and the height of the transmitter-receiver plane is h. LED’ is the projection of the LED on the ceiling; the distance between LED’ and the center of RX1 is $S_{1}$; the distance between LED’ and pin14 is $S_{2}$. Figure 1 shows that two of the biggest powers are obtained by pin11 and pin14 in RX1, and obtained by pin23 and pin24 in RX2. We can calculate angle $\beta_{1}$ and $\beta_{2}$ respectively according to the analog-to-digital conversion (ADC) value in the microcontroller control unit (MCU) in two receivers. The maximum power of receiver1 is Num11, the second value is expressed as Num12, and the third value is Num13. In the same way, receiver2 obtained Num21, Num22 and Num23, respectively. Then $\beta_{1}$ and $\beta_{2}$ can be calculated by the following:

(4) $$\left\{ \begin{array}{l} \beta_{1}=\frac{\pi }{4}\cdot \frac{Num12-Num13}{2Num11-Num12-Num13}\\ \beta_{2}=\frac{\pi }{4}\cdot \frac{Num22-Num23}{2Num21-Num22-Num23} \end{array}\right.$$

According to Equations (5) and (6), the incidence angle $\psi_{1}$ of RX1, the distance between RX1 and LED $d_{1}$ and h can be worked out. In the same way, the incidence angle $\psi_{2}$ of RX2 and the distance between RX2 and LED $d_{2}$ can be obtained.

(5) $$S_{2}=\sqrt{{\left(\frac{\sin\beta_{2}}{\sin\left(\beta_{1}+\beta_{2}\right)}\cdot l\right)}^{2}+r^{2}-2rl\frac{\sin\beta_{1}\cdot \sin\beta_{2}}{\sin\left(\beta_{1}+\beta_{2}\right)}}$$

(6) $$\left\{ \begin{array}{l} \psi_{1}=\frac{1}{2}\cdot \arcsin\sqrt{\frac{4P_{r}}{CP_{t}}\cdot \left\{{\left(\frac{\sin\beta_{2}}{\sin\left(\beta_{1}+\beta_{2}\right)}\cdot l\right)}^{2}+r^{2}-2rl\frac{\sin\beta_{1}\cdot \sin\beta_{2}}{\sin\left(\beta_{1}+\beta_{2}\right)}\right\}}\\ d_{1}=\frac{S_{2}}{\sin\psi_{1}}\\ h=\frac{S_{2}}{\tan\psi_{1}} \end{array}\right.$$

Finally, the three-dimensional position of the person who carries the LED is precisely determined with $\psi_{1}$, $\psi_{2}$, $d_{1}$, $d_{2}$ and h. Since $d_{1}$ and $d_{2}$ are the distances between the LED and a PIN, the error of the positioning distance is the annular receiver’s circle r, which is less than 0.2 m.

## 3. Results

### 3.1. The Experiment of Measuring Positioning Range

**Figure 2:**
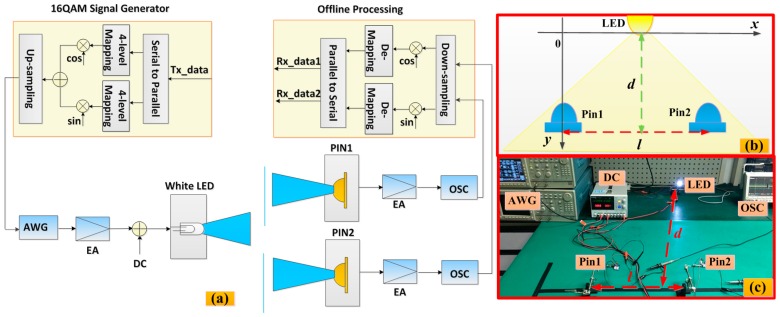
(**a**) The block diagram of the proposed VLC positioning range-testing system. EA: electrical amplifier, DC: direct current, OSC: real-time oscilloscope; (**b**) The schematic of experimental setup; (**c**) The experimental setup of measuring positioning range.

Figure 2 shows the block diagram and experimental setup for the proposed VLC positioning range-testing system respectively. In this system, the drive signals are generated by the arbitrary waveform generator (AWG) (Tektronix AWG520, Tektronix, Shanghai, China) with an offline Matlab program. After the hardware electrical amplifier, the resulting waveform coupled with the direct current is applied to the LED (Cree, XLamp XP—G Q5, CREE, Durham, NC, USA). Two commercially available PINs (HAMAMATSU S10784, HAMAMATSU, Beijing, China, effective photosensitive area: 7 mm^2^, and 0.45 A/W sensitivity with a −3 dB bandwidth of 300 MHz at 660 nm) are used for detecting the light signal. At the transmitters, up-sampling by a factor of four is employed and the original bit sequence is mapped into complex symbols of 16 QAM (quadrature amplitude modulation). In this experiment, the data rate is 10 Kbit/s, and the sample rate of the oscilloscope (Tektronix, MDO 3022, Tektronix, Shanghai, China) is 50 KS/s. The offline digital signal processing (DSP) is applied to down-sample and demodulate the sampled signal by oscilloscope (OSC). The vertical distance between the LED and receivers is d, and l is the distance between the two receivers. In order to obtain the minimum positioning range at different vertical distances, we fix the LED and change l (up to 1.2 m) on the transmitting plane.

**Figure 3:**
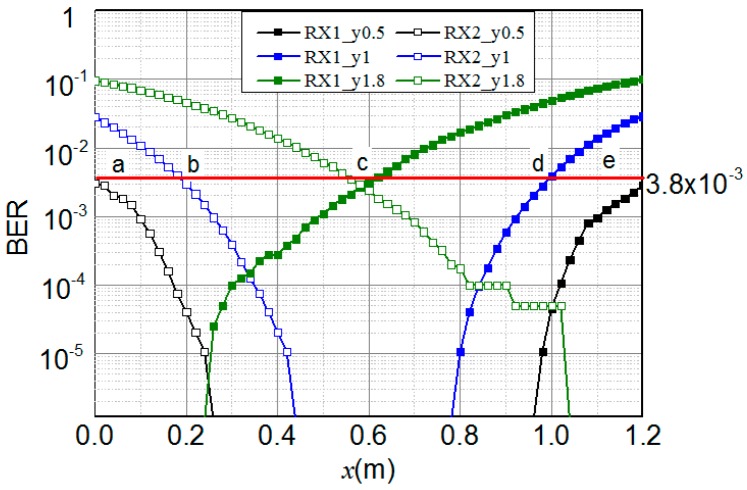
The BER distribution of two receivers when vertical distance is 0.5 m, 1 m and 1.8 m.

In the proposed VLC positioning range-testing system, we measured the bit error rate (BER) of two receivers when d is 0.5 m, 1 m and 1.8 m. Figure 3 shows the BER versus horizontal offset x while d is 0.5 m, 1 m and 1.8 m. The effective positioning condition is that the BERs of the two receivers are both below $3.8\times 10^{-3}$. As shown in Figure 3, the smaller the vertical distance is, the wider the positioning range. In addition, when d is greater than 1.8 m, only one receiver can receive the positioning signal at the same time. Positioning can be realized only when two receivers both calculate the RSS, so the experimental result means the target cannot be positioned anywhere when the distance is beyond 1.8 m. To sum up, the effective positioning range of our system is 1.8 m when the maximum distance of two receivers is 1.2 m.

### 3.2. The Experiment of Receiver’s Circle Radius and Height

**Figure 4:**
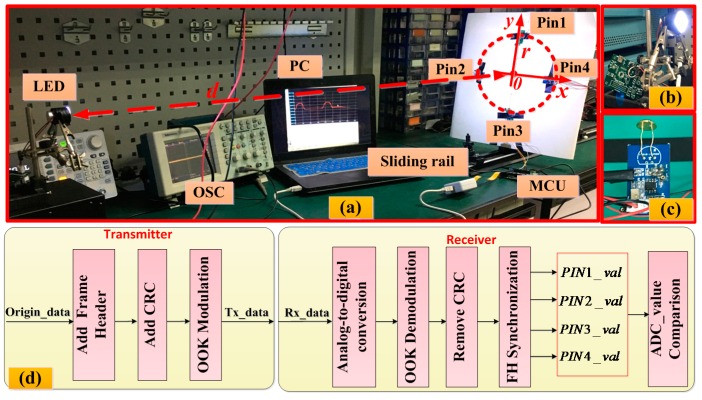
(**a**) The experimental setup of testing the receiver’s circle radius and height; (**b**) The transmitter part; (**c**) The receiver part; (**d**) The block diagram of the proposed VLC positioning system. CRC: cyclic redundancy check.

Figure 4 shows the block diagram and experimental setup to test the receiver’s circle radius and height, respectively. In this system, a microcontroller control unit (MCU) (STM32F103RCT6) in the transmitter part firstly generates the origin data, then adds a frame header to mark the beginning of each frame. A CRC (cyclic redundancy check) is also added to ensure the correctness of the data transmission. Finally, the original bit sequence is mapped into OOK (on-off keying). Four commercially available PINs (HAMAMATSU S10784, HAMAMATSU, Beijing, China, effective photosensitive area: 7 mm^2^, and 0.45 A/W sensitivity with a −3 dB bandwidth of 300 MHz at 660 nm) are used for detecting the light signal. At the receiver part, a MCU (STM32F103RCT6) is used to compare the received power of four PINs after ADC (analog-to-digital conversion), demodulation, CRC recovery and frame header (FH) synchronization. The radius of the PIN circle is r and the distance between the LED and the center of the receiver is d. Since the received power of each PIN is different, we can compare different ADC values by the MCU to calculate angles. In this system, the minimum ADC value that the MCU can distinguish is 0.1 when the ADC value is normalized. In this experiment, we fix the LED and change r (up to 0.5 m) and d (up to 1.8 m) to investigate the position performance. Moreover, in Figure 4, *O* is the center of the receiver. The sliding rail and the LED are in the same horizontal direction, so we can change the position of the sliding rail to change the x-offset of the receiver.

**Figure 5:**
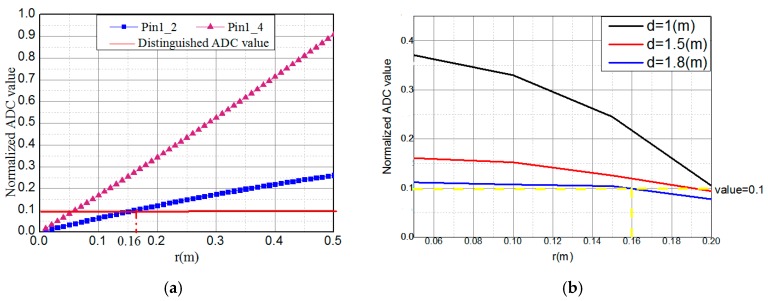
The difference of the ADC value of two adjacent PINs (**a**) at different radius of the PIN circle; (**b**) at different distance.

In Figure 5a, Pin1_2 represents the different ADC values of PIN1 and PIN2. It is obvious that the ADC differences of two adjacent PINs are the same if the center of the receiver and LED are in the same horizontal direction. In order to estimate the positioning performance in a more complex environment, we change the x-offset of the receiver. As shown in Figure 5a, with the increase of the radius, the difference of the ADC values of two adjacent PINs also gradually increases. Besides, the value of Pin1_2 is bigger than Pin1_4 because of the square relationship of the power with distance. Since the MCU can distinguish a 0.1 normalized ADC value, we obtain the minimum radius of the PINs’ circle which is 0.16 m. In Figure 5b, the ADC value difference of two adjacent PINs decreases with the increase of the height because when we change the distance, the received signal strength of each PIN changes a lot. That makes the decrease of the received signal strength the main factor in the ADC value difference. From this experiment, we obtain the maximum distance of the transmitter-receiver plane which is 1.8 m, and the maximum radius is 0.2 m. To sum up, the radius of the PIN circle is between 0.16 m and 0.2 m.

Table 1 is the estimated normalized ADC value of the difference at different x-offsets when the radius of the PIN circle is 0.2 m and the distance is 1.1 m. It is shown in the table that the power of PIN4 is the largest because it is nearest to the LED. On the contrary, PIN2 obtains the minimum value. The values of PIN1 and PIN3 are almost the same due to the symmetric distribution of the LED illumination. We are most concerned about the max_difference of the two adjacent PINs. The result shows that when the x-offset is 0.05 m, the max_difference is less than 0.1, which means the MCU cannot work out the angle. Furthermore, the max_difference is increasing with the increasing x-offset, so the system is more effective when the LED is far away from the center of the receivers.

### 3.3. The Measurement of the Positioning Error

**Figure 6:**
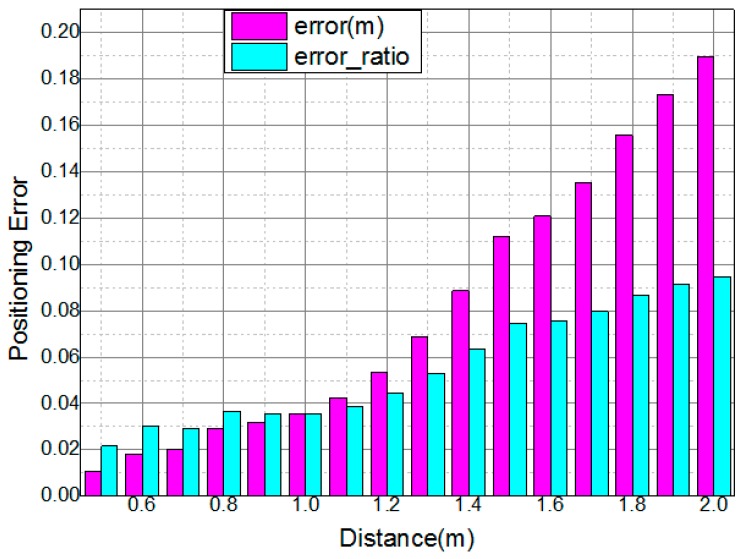
The normalized error and error ratio at different distances.

The normalized measurement error and positioning error ratio are plotted in Figure 6 in the red and green histograms, respectively. To quantify the relative error between the estimation result and the actual distance, we define the error ratio (denoted as err) as:

(7) $$err=\frac{\left|d_{measured}-d_{real}\right|}{d_{real}}$$

where $d_{measured}$ and $d_{real}$ are the distance of the calculation results of our system and the actual distance between the LED and the receivers, respectively. In Figure 6, we can see that the error ratio is mostly below 10% when the actual distances are within 2 m. In addition, the positioning error is below 0.2 m, which depends on the maximum radius of the PIN circle. According to Reference [11], the RSS positioning algorithm can achieve an accuracy of 7 cm. In the proposed indoor positioning system, we use the RSS to calculate the distance without using any lens to capture a high proportion of light. So the positioning error grows significantly with the increasing distance because of the strong power attenuation. In practice, a lens or lampshade can be fixed in front of the PINs to improve the signal-to-noise ratio (SNR), and thus the positioning accuracy of the system will be improved.

## 4. Discussion

Although the accuracy of proposed ISP based on VLC is not particularly high, it has fewer applied limitations and special functions compared with other ISPs. In Reference [14], the paper proposed an IPS using a single transmitter and a rotatable single PD with less than 3 cm of error. However, the size of the proposed device must be compact because the positioning accuracy relies on the incidence angle gain profile. In Reference [15], the target device can be positioned within centimeters with multiple PDs, but the total number of LEDs and PDs is no smaller than four. We could obtain optimal performance on the condition of sufficient SNR, but it requires a higher computational complexity compared to currently proposed methods. The proposed reversed positioning system only needs two receivers, which is more convenient and cost-efficient.

Besides, the proposed reversed VLC-based IPS is the first system with the function of personnel monitoring in some large electromagnetic-sensitive sites. Based on the proposed reversed VLC-based IPS, the security of workers will be guaranteed in nuclear power stations and aircraft carriers. Moreover, the proposed reversed positioning system does not need complex data-processing algorithms, which can improve the real-time positioning speed.

Since the effective positioning range of our system is 1.8 m, in order to locate the LED’s position in a big room, we must install multiple receivers on the ceiling. In our future research, we will study the shape of the receiver array to achieve full-range positioning in a room. Furthermore, according to Reference [16], the minimum intensity is determined by the mitigation of systematic AOA errors. In the proposed reversed IPS, the minimum intensity is determined by the ADC value that the MCU can distinguish. Since the MCU can distinguish a 0.1 normalized ADC value, we can obtain the minimum intensity according to the minimum ADC value difference of two adjacent PINs.

## 5. Conclusions

In this paper, we propose a reversed VLC indoor positioning scheme using only two receivers with annular multi-photodiodes. When the distance of the transmitter-receiver plane is less than 1.8 m and the radius of the PIN circle is between 0.16 m and 0.2 m, the effective positioning range of our system is 1.8 m within a 0.2 m positioning error. It is demonstrated that the proposed scheme can be a promising candidate for indoor positioning applications, especially in solving positioning problems of nuclear power stations.

## Figures and Tables

**Table 1:** Estimated ADC value difference at different x-offsets when the radius of the PIN circle is 0.2 m and the distance is 1.1 m.

x-Offset (m)	PIN1_Value	PIN2_Value	PIN3_Value	PIN4_Value	Max_Difference
0.05	0.7085	0.6246	0.7261	0.7905	0.082
0.1	0.6762	0.5431	0.6969	0.8388	0.2957
0.15	0.4687	0.3587	0.4232	0.9319	0.5731
0.2	0.4208	0.2808	0.3960	1	0.7192

## Abbreviations

The following abbreviations are used in this manuscript:

IPS	indoor positioning system
VLC	visible light communication
RSS	received signal strength
TOA	time of arrival
TDOA	time difference of arrival
AOA	angle of arrival
QAM	quadrature amplitude modulation
ADC	analog-to-digital conversion
MCU	microcontroller control unit
DSP	digital signal processing
CRC	cyclic redundancy check
OOK	on-off keying
